# Microbial Lipopolysaccharide Regulates Host Development Through Insulin/IGF-1 Signaling

**DOI:** 10.3390/ijms26157399

**Published:** 2025-07-31

**Authors:** Lijuan Teng, Jingyan Zhang

**Affiliations:** Shanghai Key Laboratory of Metabolic Remodeling and Health, Institute of Metabolism and Integrative Biology, Fudan University, Shanghai 200438, China

**Keywords:** host–microbe interactions, *C. elegans*, LPS, *rfaG*

## Abstract

Lipopolysaccharide (LPS), the defining outer membrane component of Gram-negative bacteria, is a potent immunostimulant recognized by Toll-like receptor 4 (TLR4). While extensively studied for its roles in immune activation and barrier disruption, the potential function of LPS as a developmental cue remains largely unexplored. By leveraging *Caenorhabditis elegans* and its genetic and gnotobiotic advantages, we screened a panel of *Escherichia coli* LPS biosynthesis mutants. This screen revealed that the loss of outer core glycosylation in the ∆*rfaG* mutant causes significant developmental delay independent of bacterial metabolism. Animals exhibited developmental delay that was rescued by exogenous LPS or amino acid supplementation, implicating that LPS triggers nutrient-sensing signaling. Mechanistically, this developmental arrest was mediated by the host FOXO transcription factor DAF-16, which is the key effector of insulin/IGF-1 signaling (IIS). Our findings uncover an unprecedented role for microbial LPS as a critical regulator of host development, mediated through conserved host IIS pathways, fundamentally expanding our understanding of host–microbe crosstalk.

## 1. Introduction

The microbiome plays crucial roles in regulating host physiology, with microbes implicated in host diseases ranging from metabolic syndrome to neurodegeneration. Among abundant microbial molecules and metabolites, lipopolysaccharide (LPS), a hallmark component of Gram-negative bacterial outer membranes, is secreted via outer membrane vesicles (OMVs) and potently activates mammalian innate immunity [1,2,3]. LPS comprises three domains: the hydrophobic lipid A, inner core oligosaccharide, and outer core O-antigen. Lipid A, the conserved endotoxic moiety, anchors the LPS molecule to the bacterial outer membrane and drives innate immune activation via Toll-like receptor 4 (TLR4) in mammals. The O-antigen determines serotype specificity and resistance to host defenses, while the inner core oligosaccharide containing 3-deoxy-D-manno-oct-2-ulosonic acid (KDO) is essential for the synthesis of bioactive lipid A and influences host–pathogen interactions [4,5,6,7,8]. Structural modifications to the outer core (e.g., acetylation) further tune LPS immunogenicity and environmental adaptability [9]. This tripartite architecture differentiates Gram-negative bacteria from Gram-positive species. In contrast to Gram-negative bacteria, Gram-positive bacteria lack LPS but possess teichoic acid-embedded peptidoglycan [10,11]. LPS acts as a pathogen-associated molecular pattern (PAMP), triggering oxidative stress, intestinal barrier disruption, and immune signaling in metazoan hosts [12,13,14,15,16,17,18,19]. Paradoxically, while LPS is best characterized as an inflammatory toxin, its potential role as a developmental cue remains unexplored.

The nematode *Caenorhabditis elegans* offers unparalleled advantages for dissecting host–microbe interactions: genetic tractability, transparent anatomy, and gnotobiotic control enable precise analysis of microbes [20,21,22]. A simplified yet conserved intestinal architecture featuring microvilli and antimicrobial peptide production mirrors human gut physiology [23,24,25,26]. Additionally, the clear genetic background and defined developmental cell lineage enable the tracking of gene functions at different developmental phases, while gnotobiotic culturing allows systematic investigation of the role of bacterial strains and mutants [22,27,28,29]. These features provide a robust foundation for understanding the genetic mechanisms underlying host–microbe interactions [30].

*C. elegans* encounters diverse pathogens whose invasion mechanisms illuminate universal virulence principles, and it also serves as a powerful model organism for dissecting host–pathogen interactions due to its genetic tractability, transparent anatomy, short lifecycle, and conserved innate immune pathways. Despite lacking adaptive immunity, *C. elegans* mounts robust defenses against diverse pathogens (bacteria, fungi, and viruses) through evolutionarily conserved mechanisms. Prior studies of LPS have focused predominantly on its cytotoxic effects [16,17,19], overlooking potential functions in host development and metabolism. Here, we bridge this gap through a genetic screening of *E. coli* LPS biosynthesis mutants. By employing a high throughput platform, we identified the *E. coli* mutant ∆*rfaG*, which is defective in outer core glycosylation synthesis, as a key regulator of *C. elegans* development. This developmental delay is independent of bacterial metabolism but is rescued by exogenous LPS or amino acids, implicating that LPS modulates host nutrient-sensing signaling. Mechanistically, we establish that this developmental delay is mediated by the host FOXO transcription factor DAF-16 in insulin signaling (IIS). Our work uncovers LPS as an essential cue that regulates host development through conserved nutrient-sensing pathways, revealing an unexpected crosstalk between microbial surface architecture and host developmental programming.

## 2. Results

### 2.1. Establishment of a High-Throughput Screening Platform for C. elegans Development

**Figure 1:**
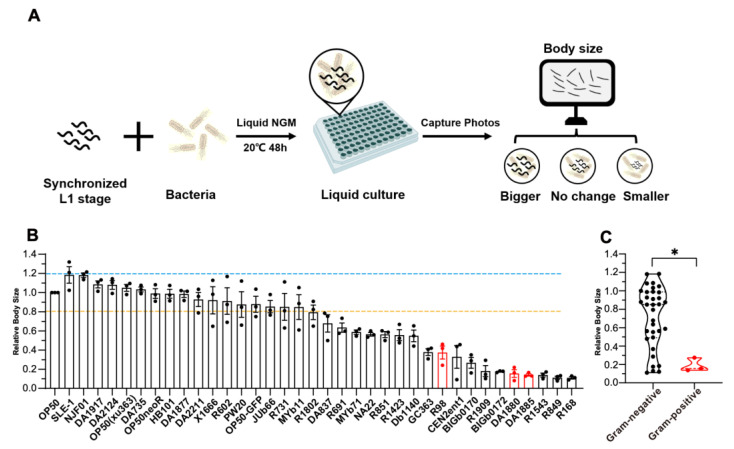
High-throughput screen identifying Gram-negative bacteria as potent promoters of *C. elegans* development. (**A**) Workflow for liquid culture developmental screening. Synchronized L1 larvae were co-cultured with bacterial suspensions. Bright-field images were captured using the Invitrogen EVOS FL imaging system, and body size was analyzed using ImageJ software, version1.54K. (**B**) Bar graph comparing *C. elegans* developmental rate across bacterial isolates. Data were normalized to the mean body size of nematodes fed wild-type *Escherichia coli* OP50 (control; dashed line). Body size outside this range exhibits significant differences. (**C**) Body size distribution of worms fed Gram-negative (dark) versus Gram-positive bacteria (red). * *p* < 0.05 (unpaired two-tailed *t*-test). Data represent mean ± SEM.

To systematically investigate the impact of bacteria on host development and metabolism, we developed a high-throughput assay using animal body size as a quantitative proxy for developmental progression (Figure 1A). We employed this platform to screen bacterial strains for their ability to modulate host development rate and body size (Table 1). Animals fed different bacterial strains displayed distinct developmental outcomes and varied body size, underscoring the complexity of host–microbe interactions (Figure 1B). Notably, animals fed Gram-negative bacteria (dark-shaded column) exhibited significantly larger body sizes compared to those fed Gram-positive species (red column) (Figure 1C), suggesting potentially enhanced nutritional composition or specific signaling molecules from Gram-negative microbes.

### 2.2. The LPS Biosynthesis Mutant *Δ*rfaG Impairs C. elegans Development

**Figure 2:**
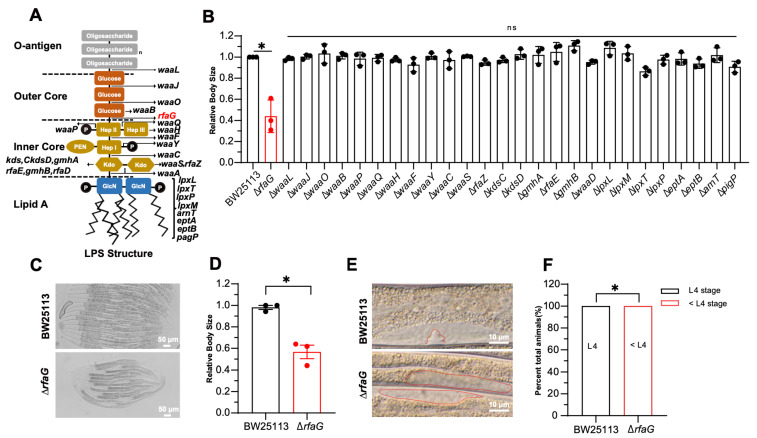
The Δ*rfaG* mutant impairs *C. elegans* development. (**A**) Schematic of *E. coli* LPS structure. (**B**) Body size of *C. elegans* fed LPS biosynthesis mutants (ns, not significant; * *p* < 0.05 vs. BW25113 control). (**C**,**D**) Representative images and quantification of worms fed Δ*rfaG* (scale bar: 50 μm). (**E**,**F**) Developmental stage of *C. elegans* grown on ∆*rfaG* for 48 hour. Vulval morphology (Christmas tree-like structure, top) and germline (bottom) were visualized by red line(scale bar: 10 μm). Data are mean ± SEM (Data are from 3 independent biological replicates, more than 25 worms per group, and 3 independent experiments) (* *p* < 0.05 (two-tailed *t*-test)).

Given the structural divergence in bacterial cell walls, specifically the presence of lipopolysaccharide (LPS) in Gram-negative outer membranes, we hypothesized that LPS could act as a key modulator of *C. elegans* development. To test this hypothesis, we systematically screened a panel of *E. coli* LPS biosynthesis mutants. To exclude the pathogenic effects, we selected the *E. coli* Keio single-gene deletion library, which is non-pathogenic to *C. elegans*. The *Escherichia coli* LPS comprises three domains: lipid A, core polysaccharide, and O-antigen [31] (Figure 2A). We found that only the Δ*rfaG* mutant induced significantly smaller body size in *C. elegans* (Figure 2B–D). The *rfaG* gene encodes a glycosyltransferase critical for core polysaccharide biosynthesis [12,32,33,34]. To determine whether the reduced body size reflected developmental delay, we examined the developmental stage of *C. elegans*. Animals fed *E. coli* BW25113 reached the L4 stage, marked by the characteristic Christmas tree-like vulval morphology after 48 h, whereas animals fed the Δ*rfaG* mutant exhibited significant delay and failed to progress to the L4 stage (Figure 2E,F). Together, these results identify the Δ*rfaG* mutation as a specific inducer of *C. elegans* developmental delay, and this phenotype is independent of broader LPS biosynthesis defects or general bacterial fitness impairment.

### 2.3. LPS Structure Mediates *Δ*rfaG-Induced Developmental Delay

**Figure 3:**
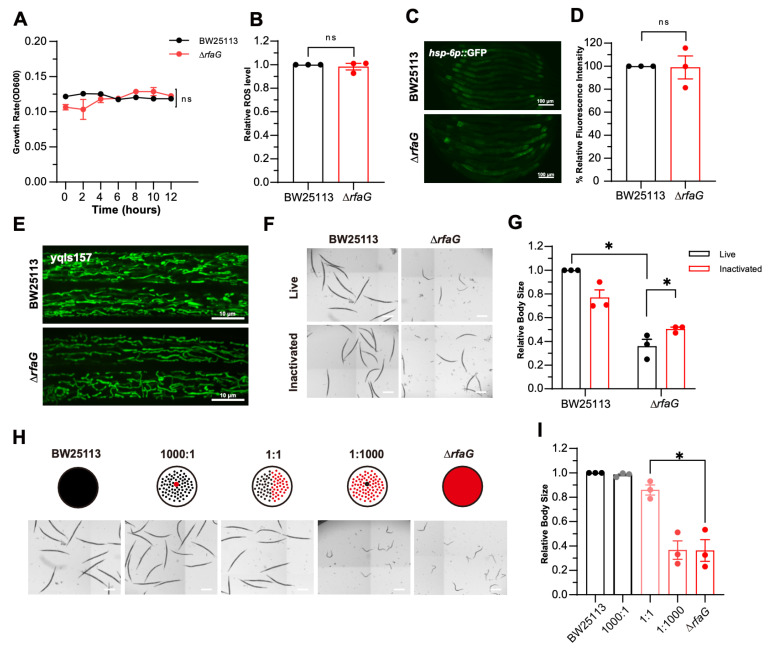
Microbial LPS drives host developmental delay. (**A**) Bacterial growth rates of BW25113 and Δ*rfaG*. (**B**) Reactive oxygen species (ROS) levels in BW25113 vs. ∆*rfaG*, measured via H2DCFDA fluorescence. (**C**,**D**) Representative images and quantitative analysis of *hsp-6p*::GFP expression in *C. elegans* fed with BW25113 vs. ∆*rfaG*(Scale bar: 100 μm). (**E**) Representative images of mitochondria in *C. elegans* fed with BW25113 vs. ∆*rfaG* (Scale bar: 10 μm). (**F**,**G**) Metabolically inactivated (PFA-fixed) Δ*rfaG* rescues host developmental delay (scale bar: 500 μm). (**H**,**I**) Supplementation wild-type BW25113 restores the host developmental delay (scale bar: 500 μm). Data are mean ± SEM (Data are from 3 independent biological replicates, more than 20 worms per group, and 3 independent experiments) (ns, not significant; * *p* < 0.05 (two-tailed *t*-test)).

To dissect the mechanism underlying ∆*rfaG*-induced host developmental delay, we first assessed whether bacterial growth contributed to the host developmental delay. Both wild-type BW25113 and Δ*rfaG* strains exhibited no significant growth due to the limited nutrients in the medium, indicating that the host developmental delay was not attributable to a bacterial growth defect (Figure 3A). Deletion of LPS synthesis genes has been reported to increase bacterial reactive oxygen species (ROS) levels and oxidative stress [35]. ROS are reported to play roles in various biological processes like DNA replication and protein homeostasis [36,37,38,39]. To determine whether ROS levels induced *C. elegans* developmental delay, we measured bacterial ROS levels using the 2′,7′-dichlorodihydrofluorescein diacetate (H2DCFDA). There were no significant differences in ROS levels in the bacteria BW25113 and Δ*rfaG* (Figure 3B). The mitochondrial unfolded protein response (UPR^mt^) has been reported to be closely associated with a variety of oxidative stresses [40,41]. However, Δ*rfaG* did not activate UPR^mt^ in *C. elegans* (Figure 3C,D). Mitochondrial morphology (visualized using *yqIs157*) also retained normal tubular structures (Figure 3E), further excluding oxidative stress as a driver of host developmental delay. The structure of the mitochondria in *C. elegans* fed Δ*rfaG* maintained normal tubular morphology (Figure 3E). These findings suggest that the Δ*rfaG*-induced developmental delay in *C. elegans* is not associated with oxidative stress.

To elucidate whether the effects of Δ*rfaG* are dependent on bacteria metabolism, we treated the bacteria with paraformaldehyde (PFA) to inactivate their metabolic activity [42]. Remarkably, the PFA-inactivated Δ*rfaG* exhibited the *C. elegans* developmental defect (Figure 3F,G). This result suggests that the LPS structure in Δ*rfaG*, rather than live bacteria metabolites, mediates delayed development in *C. elegans*.

Next, we determined the threshold at which wild-type BW25113 could rescue the growth of *C. elegans* fed on Δ*rfaG*. We titrated wild-type BW25113 into Δ*rfaG* with varying concentrations. Mixing small amounts of wild-type BW25113 with ∆*rfaG* did not alleviate *C. elegans* developmental delay; ≥50% wild-type bacteria restored normal development of *C. elegans* (Figure 3H,I). This demonstrates that intact LPS architecture is critical for *C. elegans* growth, with no compensatory capacity below this threshold. Collectively, our findings suggest that structural changes in LPS, rather than bacterial metabolites, drive ∆*rfaG*-induced host developmental delay, providing crucial insights into the role of LPS in host–microbe interactions.

### 2.4. The *Δ*rfaG Mutant Induced Developmental Defects via DAF-16

**Figure 4:**
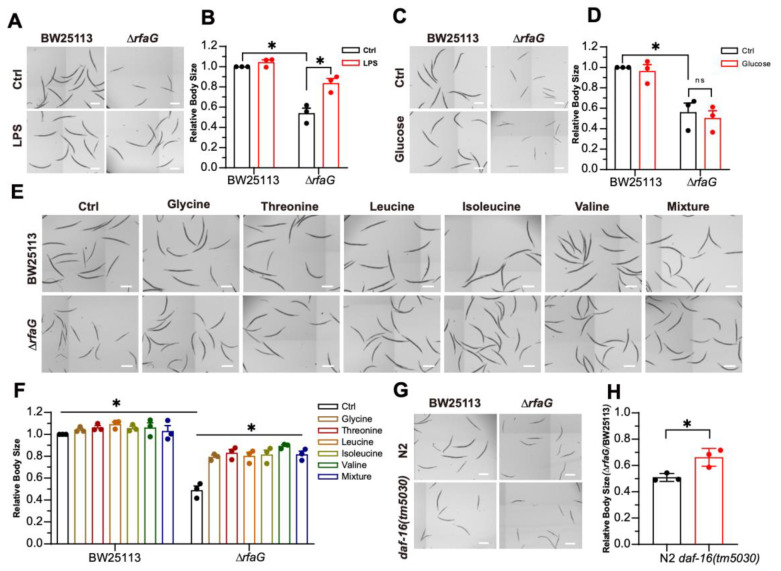
Exogenous LPS and amino acids rescue Δ*rfaG*-induced developmental defects mediated by DAF-16. (**A**,**B**) LPS rescues Δ*rfaG*-induced developmental delay(scale bar: 500 μm). (**C**,**D**) Glucose supplementation fails to rescue the developmental defect. (**E**,**F**) Amino acid supplementation restores animal growth (scale bar: 500 μm,* *p* < 0.05 (one-way-ANOVA). (**G**,**H**) *daf-16* loss-of-function mutants bypass Δ*rfaG*-induced developmental defect(scale bar: 500 μm). Data are mean ± SEM (Data are from 3 independent biological replicates, more than 20 worms per group, and 3 independent experiments) (ns, not significant; * *p* < 0.05 (two-tailed *t*-test)).

To determine whether LPS directly contributes to ∆*rfaG*-induced developmental defects in *C. elegans*, we supplemented Δ*rfaG* cultures with purified LPS, which fully rescued the developmental delay in *C. elegans* induced by Δ*rfaG* (Figure 4A,B). This result provides direct evidence for the essential role of bacterial LPS in promoting the growth of *C. elegans*. LPS influences the intestinal nutrient absorption in *C. elegans* [20], so we tested whether the Δ*rfaG*-induced developmental delay was caused by nutrient deficiency. We supplemented *C. elegans* with several nutrients. Supplementation of glucose did not alleviate the developmental delay of *C. elegans* induced by Δ*rfaG* (Figure 4C,D). We further tested a set of amino acids (glycine, threonine, leucine, isoleucine, and valine) for their ability to restore host growth (Figure 4E,F), suggesting amino acid scarcity underlies Δ*rfaG*-induced host developmental delay.

The insulin/IGF-1 signaling (IIS) integrates nutrient status via FOXO transcription factor DAF-16 and plays a crucial role in regulating developmental arrest and gene expression [43,44]. The FOXO transcription factor DAF-16, a downstream effector of the IIS pathway, is responsible for integrating stress signals and translocating from the cytoplasm to the nucleus [45]. To determine if DAF-16 mediates developmental arrest in *C. elegans* induced by Δ*rfaG*, we examined the development of *daf-16(tm5030)* animals fed on Δ*rfaG*; *daf-16(tm5030)* mutants fed on Δ*rfaG* bypassed developmental arrest (Figure 4G,H), indicating that DAF-16 mediates developmental arrest induced by Δ*rfaG*. Together, these data indicate a cascade wherein LPS limits host amino acid availability, exogenous LPS or amino acids alleviate the developmental delay, and the insulin-like signaling (IIS) pathway mediates the developmental delay.

**Figure 5:**
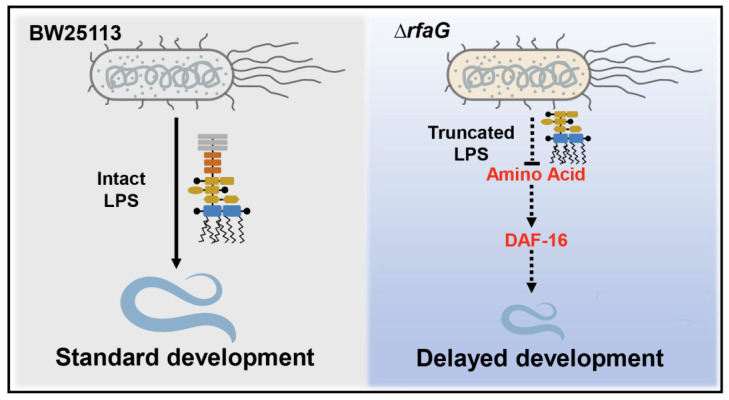
Schematic model for ∆*rfaG*-induced developmental delay in *C. elegans* via FOXO/DAF-16.

In summary, our findings demonstrate that *E. coli* LPS is essential for *C. elegans* development. The Δ*rfaG* mutant, with a truncated LPS structure, induces developmental defects by limiting host amino acid availability. These developmental defects are mediated by DAF-16, the FOXO transcription factor in the insulin/IGF-1 signaling pathway, and rescued by exogenous LPS or amino acids, highlighting LPS structure as a critical determinant of host–microbe metabolic crosstalk (Figure 5).

## 3. Discussion

Previous studies have emphasized LPS as a pro-inflammatory endotoxin that induces cellular damage and immune responses [14,19,46]. However, our work uncovered an unrecognized role of bacterial LPS in *C. elegans* development. We demonstrated that Δ*rfaG* with a structural defect in LPS profoundly disrupts *C. elegans* development. This finding challenges the traditional paradigm that bacterial metabolic outputs (e.g., nutrient provisioning) are the drivers of *C. elegans* developmental progression. Instead, our data positions LPS architecture as a critical structural signal in host–microbe crosstalk. This novel finding expands our understanding of the molecular mechanisms underlying host–microbe interactions in *C. elegans*.

A pivotal innovation of this study lies in disentangling LPS’s structural role from bacterial metabolic activity. The rescue of developmental delay by paraformaldehyde (PFA)-fixed Δ*rfaG* (Figure 3G) unequivocally demonstrates that LPS integrity—not live bacteria or secreted metabolites—is essential for normal development. Future studies could explore whether homologs of these receptors mediate LPS recognition in *C. elegans*.

LPS, a structural component of the bacterial cell wall, serves as a crucial signal molecule [7]. The rescue of Δ*rfaG*-induced defects by exogenous amino acids (Figure 4E,F) points to a novel mechanistic link between LPS structure and host nutrient utilization. We hypothesize that intact LPS facilitates amino acid uptake or signaling, possibly by maintaining intestinal barrier integrity or modulating nutrient-sensing pathways. This mirrors findings in mammals, where LPS dysbiosis impairs amino acid absorption and triggers metabolic stress [46]. Intriguingly, the insulin/IGF-1 signaling (IIS) pathway—a central regulator of nutrient sensing and longevity in *C. elegans*—emerged as a key player, as *daf-16* mutants bypassed developmental arrest (Figure 4G,H). This parallels studies where IIS pathway components, including DAF-16, mediate lifespan extension under dietary restriction or protease inhibition [44,45], suggesting a conserved nexus between microbial signals, nutrient availability, and developmental plasticity. Given that amino acids can rescue the developmental defects induced by Δ*rfaG*, LPS may affect the uptake or utilization of amino acids in *C. elegans*. Further research is needed to explore this potential relationship between LPS and amino acids.

The suppression of Δ*rfaG*-induced defects in *daf-16* mutants highlights DAF-16’s dual role as a stress-responsive transcription factor and a developmental checkpoint. While DAF-16 is best known for its role in longevity under stress, our work expands its function to include developmental modulation in response to microbial cues. This aligns with recent discoveries that DAF-16 integrates environmental signals, such as oxidative stress and pathogen exposure, to regulate organismal fitness. Future investigations should delineate whether LPS directly modulates DAF-16 nuclear translocation or collaborates with upstream IIS components like AGE-1/ PI3K.

Notably, our findings contrast with studies where LPS triggers neuroinflammation and behavioral deficits in mammals, underscoring the context-dependent nature of LPS signaling. In *C. elegans*, the absence of canonical TLR4 homologs may explain why LPS acts as a developmental cue rather than an inflammatory trigger. This divergence highlights the evolutionary plasticity of LPS–host interactions and positions *C. elegans* as a unique model to study LPS’s non-canonical roles.

## 4. Materials and Methods

### 4.1. C. elegans Strains and Maintenance

All nematode strains in this study were maintained on nematode growth medium (NGM) using standard protocols [47]. NGM was prepared as previously described [47] (1.7% agar, 0.3% NaCl, 0.25% peptone, 1 mM CaCl_2_, 1 mM MgSO_4_, 5 μg/mL cholesterol, and 25 mM KH_2_PO_4_ buffer pH 6.0). Gravid adults were treated with 5% hypochlorite solution and 0.5 M NaOH for 5 min to release embryos, which were washed 5 times with M9 buffer (22 mM KH_2_PO_4_, 42 mM Na_2_HPO_4_, 85 mM NaCl, and 1 mM MgSO_4_), followed by incubation in liquid NGM at 20 °C for 48 h to obtain synchronized L1 larvae. Synchronized L1 larvae were obtained via hypochlorite treatment of gravid adults, followed by incubating in liquid NGM at 20 °C for 48 h. The following strains were used in the study: wild-type N2, SJ4100 [*zcls13* (*Phsp-6*::GFP)], yqIs157 [(P*y37a1b.5*::mito-GFP), and XV92[*daf-16*(tm5030)].

### 4.2. Bacterial Strains and Maintenance

*E. coli* BW25113 (WT) was cultured in Lysogeny Broth (LB) medium at 37 °C. *E. coli* single-gene deletion mutants from the Keio collection were grown in LB medium supplemented with 50 μg/mL kanamycin at 37 °C.

### 4.3. Reagents

Stock solutions were prepared as follows: LPS (5 mg/mL), glucose (350 mM), glycine (1.7 M), threonine (280 mM), leucine (64 mM), isoleucine (190 mM), and valine (170 mM); stored at −20 °C. Paraformaldehyde (PFA, 80096618) was purchased from Sinopharm (Beijing, China) and stored as a 4% solution at 4 °C. Liquid NGM was prepared as standard NGM without agar.

### 4.4. E. coli LPS Synthesis Mutants Screen in Liquid Culture System

*C. elegans* embryos were harvested from gravid worms fed *E. coli* BW25113. Adults were treated with alkaline hypochlorite solution and incubated for 20 h in M9 buffer at 20 °C. Synchronized L1 larvae (≈25 per well) in 10 μL M9 buffer were aliquoted into 96-well plates. Overnight cultures of *E. coli* LPS biosynthesis mutants were centrifuged (3000× *g*, 30 min), washed, and resuspended in liquid NGM to OD_600_ = 8.0. A total of 10 μL of bacterial suspension was added to each well containing larvae, followed by 50 μL liquid NGM. Plates were sealed and incubated statically at 20 °C for 48 h. Worms were then immobilized with M9 buffer containing 1 mM levamisole, imaged using an Invitrogen EVOS FL microscope (Thermo Fisher, Waltham, MA, USA), and body size was quantified using ImageJ software, version1.54K [48].

### 4.5. Assessment of Bacterial Growth Rate

A total of 10 μL of bacterial culture and 60 μL of liquid NGM were added to 96-well plates and incubated at 220 rpm at 20 °C. A total of 10 μL of bacterial culture was diluted to 100 μL to measure OD600. OD_600_ was measured every 2 h for 12 h using a microplate reader (TECAN, Mannedorf, Switzerland). A total of 10 μL bacterial culture was diluted with 90 μL liquid NGM to measure OD_600_.

### 4.6. Assessment of Bacterial ROS Levels

Logarithmic-phase bacterial cultures were centrifuged (3000× *g*, 10 min), washed, and resuspended in PBS (pH 7.4) to OD_600_ ≈ 0.5. Bacteria were incubated with 10 μM H_2_DCFDA (2′,7′-dichlorodihydrofluorescein diacetate; Invitrogen, Carlsbad, CA, USA, Cat. No. D399) in the dark at 37 °C for 1 h. Fluorescence intensity (excitation 492 nm, emission 535 nm) was measured in a black-walled 96-well plate using a microplate reader.

### 4.7. Paraformaldehyde Treatment of Bacteria

Overnight bacterial cultures were treated with 0.5% (*v*/*v*) PFA (final concentration) and incubated with shaking (220 rpm) at 37 °C for 1 h. Fixed bacteria were centrifuged (3000× *g*, 30 min) and washed five times with sterile LB medium. PFA-killed pellets were resuspended in liquid NGM for assays.

### 4.8. Bacterial Mixing Assay

Overnight cultures of *E. coli* BW25113 (WT) and Δ*rfaG* were centrifuged (3000× *g*, 10 min), washed, and resuspended in liquid NGM to OD_600_ = 8.0. Bacteria were mixed at WT: Δ*rfaG* ratios of 1000:1, 1:1, and 1:1000. Synchronized L1 larvae, bacterial mixtures, and liquid NGM were combined in 96-well plates. Plates were sealed, incubated at 20 °C with shaking (220 rpm) for 48 h, and imaged.

### 4.9. Statistical Analysis

Statistical analyses were performed using GraphPad Prism 8.0.2 (GraphPad Software) and Fiji (ImageJ). Data are presented as mean ± SEM. Significance was determined by unpaired two-tailed Student’s *t*-test (two groups) or one-way ANOVA with Tukey’s post hoc test (≥3 groups). Significance levels: * *p* < 0.05, ** *p* < 0.01, and ns: not significant. A minimum of three independent biological replicates were performed.

## Figures and Tables

**Table 1:** Bacterial strains of the screening.

Strain	Gram	Genus
OP50	negative	*Escherichia*
SLE-1	negative	*Escherichia*
NJF01	negative	*Escherichia*
DA1917	negative	*Escherichia*
DA2124	negative	*Escherichia*
OP50(xu363)	negative	*Escherichia*
DA735	negative	*Escherichia*
OP50-NeoR	negative	*Escherichia*
HB101	negative	*Escherichia*
DA1877	negative	*Comamonas*
DA2211	negative	*Escherichia*
X1666	negative	*Escherichia*
R602	negative	*Acidovorax*
PW20	negative	*Escherichia*
OP50-GFP	negative	*Escherichia*
JUb66	negative	*Lelliottia*
R731	negative	*Sphingomonas*
MYb11	negative	*Pseudomonas*
R1802	negative	*Acidovorax*
DA837	negative	*Escherichia*
R691	negative	*Chryseobacterium*
MYb71	negative	*Ochrobactrum*
NA22	negative	*Escherichia*
R851	negative	*Sphingomonas*
R1423	negative	*Chryseobacterium*
Db1140	negative	*Serratia*
GC363	negative	*Escherichia*
R98	positive	*Agrococcus*
CEN2ent1	negative	*Enterobacter*
BIGb0170	negative	*Sphingobacterium*
R1909	negative	*Chryseobacterium*
BIGb0172	negative	*Comamonas*
DA1880	positive	*Bacillus*
DA1885	positive	*Bacillus*
R1543	negative	*Pedobacter*
R849	negative	*Azospirillum*
R168	negative	*Azospirillum*

## Data Availability

The original contributions presented in the study are included in the article, further inquiries can be directed to the corresponding authors.
